# Supplementary material for: Application and renovation evaluation of Dalian’s industrial architectural heritage based on AHP and AIGC
Source: PLoS One. 2024 Oct 31;19(10):e0312282. doi: 10.1371/journal.pone.0312282 (PMC11527283; doi:10.1371/journal.pone.0312282)
Supplement: Attachment — Submitted filename: Response to Reviewers.doc [file pone.0312282.s001.doc]

Response letter

Journal: PLOS ONE

Title: Application and Renovation Evaluation of Dalian's Industrial Architectural Heritage Based on AHP and AIGC

The authors would like to thank the reviewer for his/ her valuable, detailed, and constructive comments, which helped us further improve the quality of the submitted manuscript. All the comments and suggestions are carefully addressed in the revised manuscript.

The authors tried their level best to address all the queries, and the point-to-point response is given below:

Response to reviewer 1 comments:

Reviewer#1, Concern # 1: The abstract is not specific enough and should include some specific numerical conclusions.

Reply: Thanks for your comments. We have revised the abstract to include specific numerical conclusions for greater clarity and completeness. Here is the updated abstract: This paper takes the example of industrial architectural heritage in Dalian to explore design scheme generation methods based on generative artificial intelligence (AIGC). The study compares the design effects of three different tools using the Analytic Hierarchy Process (AHP). It first establishes the key indicator weights for the renovation of industrial architectural heritage, with the criterion layer weights as follows: building renovation 0.230, environmental landscape 0.223, economic benefits 0.190, and socio-cultural value 0.356. Among the goal layer weights, the highest weight is for the improvement of living quality at 0.129, followed by resident satisfaction at 0.096, and educational and display functions at 0.088, while the lowest is for renovation costs at only 0.035.The design schemes are generated using Stable Diffusion, Mid Journey, and Adobe Firefly tools, and evaluated using a weighted scoring method. The results show that Stable Diffusion excels in overall image control, Mid Journey demonstrates strong artistic effects, while Adobe Firefly stands out in generation efficiency and ease of use. In the overall score, Stable Diffusion leads the other two tools with scores of 6.1 and 6.3, respectively. Compared to traditional design processes, these tools significantly shorten the design workflow and cycle, improving design quality and efficiency while also providing rich creative inspiration. Overall, although current generative artificial intelligence tools still have limitations in understanding human emotions and cultural differences, with continuous technological iteration, this method is expected to play a larger role in the design field, offering more innovative solutions for the renovation of industrial architectural heritage.

________________________________________

Reviewer#1, Concern # 2: The research background is not clear. It mentions that the traditional design method is cumbersome and artificial intelligence will be faster. However, there are many steps and links in the design field. What aspects are being improved?

Reply: Thanks for your comments. We have clarified some of the content in the research background, and here is the revised version: Due to the complexity of the design process, even minor modifications often require designers to return to certain stages of work for adjustments (Schetinger, V., 2023; Mortazavi, A., 2023). For example, slight changes in layout may necessitate reworking sketches, models, and renderings, leading to significant delays and increased costs. Additionally, clients often request multiple design options for a single project, further increasing the overall workload. While the demand for alternatives is crucial for enhancing client satisfaction, it can also exacerbate the challenges within the design workflow. Consequently, the complexity of the design process and the pressure of market competition limit project efficiency and hinder designers from fully expressing their creativity (Chen J, 2023; Huang J, 2023).

Therefore, these traditional methods face several key issues that need improvement: 1. Time and efficiency, 2. Repetitive labor, 3. Demand for multiple design options, 4. Significant investment of human and material resources. The emergence of generative artificial intelligence (AIGC) provides transformative opportunities to simplify the traditional design process (Ooi, K.-B., 2023; Batarseh, F., 2020). By introducing generative AI, the role of designers gradually shifts from "creators" to "decision-makers," fundamentally altering the conception and development of architectural design. The design process is inherently creative, and the ability to encode and simulate design experiences through AI algorithms is particularly significant.

Specifically, AIGC offers transformative opportunities to simplify the traditional design process and effectively address the challenges faced in architectural design. AIGC can automate the generation of preliminary design proposals, allowing designers to quickly obtain multiple options by simply inputting keywords, thereby significantly shortening the design cycle. Additionally, AIGC can automatically complete many traditional design steps, such as concept generation, color matching, and structural optimization, reducing repetitive labor for designers who can now adjust parameters without starting from scratch. With AIGC, designers can also generate multiple options for clients to choose from in a short time, enhancing client satisfaction and optimizing overall resource investment. AIGC tools typically integrate various AI technologies, such as deep learning and edge detection algorithms, to quickly produce high-quality renderings, thereby reducing reliance on specialized software and human resources. Through these improvements, designers can focus more on creative exploration, enhance work efficiency, and drive the development of design innovation. Designers can leverage these AIGC tools to enhance their creativity and explore a wider range of possibilities than traditional methods allow. For instance, designers no longer need to spend days or weeks iterating on sketches and models; instead, they can utilize AI to generate visual representations of their ideas in a short time. By rapidly generating multiple design iterations, AIGC enables designers to engage in broader exploration and experimentation, resulting in innovative solutions that may not have been previously considered.

________________________________________

Reviewer#1, Concern # 3: The literature review needs to be further refined. At present, it is more about introducing software and tools. However, original research papers are not just about introducing tools, but should also clarify the pain points of current research and the solutions of existing scholars.

Reply: Thanks for your comments. We have added the pain points of current research and existing scholars' solutions in the literature review section of the article, as detailed below:

Despite the significant advantages of generative artificial intelligence, several limitations and challenges remain to be addressed. One major limitation is the difficulty of incorporating cultural and emotional aspects into AI-generated designs. Current AI tools often lack a comprehensive understanding of the cultural and emotional significance of heritage sites, which may result in designs that are aesthetically pleasing but culturally inappropriate. Researchers like Li et al. (2023) are working to integrate cultural heritage data into AI algorithms to enhance their sensitivity to these aspects. This includes training AI models on datasets that incorporate cultural and historical information, enabling them to generate designs that are visually appealing and culturally relevant.

Another challenge is the need for interdisciplinary collaboration in the application of generative artificial intelligence in industrial heritage(Xiao et al., 2024). Protection and renewal projects often require input from multiple stakeholders, including architects, historians, engineers, and community members. Effective communication and collaboration are crucial for the success of such projects. Generative artificial intelligence tools can facilitate this collaboration by providing detailed visualizations and simulations, which aid in sharing and discussing ideas among stakeholders(Jiang et al., 2023). This helps ensure that all perspectives are considered and that the final design meets the needs and expectations of all relevant parties.

Additionally, issues of data quality and availability pose challenges. Generative artificial intelligence models require a substantial amount of high-quality data to operate effectively. This includes data on existing structures, materials, and historical records. In many cases, this data may be incomplete or unavailable, limiting the effectiveness of AI tools. Researchers are exploring various approaches to address this issue, including employing advanced data collection techniques and developing more sophisticated AI models capable of handling incomplete or imperfect data(Epstein et al., 2023).

The potential benefits of generative artificial intelligence in the protection and renewal of industrial heritage are significant(Nair, 2024). By automating many tasks traditionally performed by human designers, these tools can reduce the time and cost of protection projects, improve design quality and accuracy, and provide rich creative inspiration(Haze, 2023). Furthermore, the ability of generative artificial intelligence to rapidly and efficiently generate multiple design alternatives helps ensure that the best solution is found for each project.

________________________________________

Reviewer#1, Concern # 4: 2.2 Current Research Status of Industrial Heritage Renewal mentions a lot of Chinese policies and lacks an international perspective.

Reply: Thanks for your comments. We have supplemented the literature review section of the article with Chinese policies and included current international policies, as detailed below:

In China, regulations such as the "National Industrial Heritage Management Measures," "Implementation Plan for Promoting the Protection and Utilization of Industrial Heritage in Old Industrial Cities," and "Interim Measures for the Management of Industrial Heritage in Dalian" emphasize the evaluation, protection, and reuse of industrial heritage. Studies like the "Satisfaction Evaluation of Post-Reuse of Old Industrial Sites" and the "Evaluation Study on the Transformation Plan of Old Industrial Buildings with Functional Replacement" propose detailed evaluation and implementation strategies(Han, 2023) . Combined with the "Comprehensive Evaluation of the Protection and Reuse of Modern Architectural Heritage" and the "National Hygienic City Evaluation System Study," a comprehensive evaluation system has been formed (Jiang, 2016).Tian Wei et al.(2022) used an old industrial building in Tianjin as a case study, applying the entropy weight method to determine index weights, and then using the fuzzy comprehensive evaluation method to establish an evaluation model to measure satisfaction. Yang Ye et al.(2021) analyzed three cases of industrial heritage in Shanghai, based on the concept of sharing, and sorted out design indicators related to the indoor and outdoor environment from both design and operation perspectives.

Internationally, studies like "Developing a Multi-Criteria Model for the Protection of Built Heritage from the Aspect of Energy Retrofitting," "Developing a Landscape Sustainability Assessment Model Using an Analytic Hierarchy Process in Korea," and "Multicriteria Decision Tool for Sustainable Reuse of Industrial Heritage into its Urban and Social Environment" utilize the Analytic Hierarchy Process (AHP) to evaluate industrial heritage through multi-criteria decision-making and sustainable development. Additionally, "The Research on Regional Conservation Planning of Urban Historical and Cultural Areas Based on GIS" and "Application of the Analytic Hierarchy Process to Developing Sustainability Criteria and Assessing Heritage and Modern Buildings in the UAE" explore GIS technology in heritage protection, offering robust guidance for the preservation and renewal of industrial heritage.

________________________________________

Reviewer#1, Concern # 5: Considering the complexity and diversity of urban environments, how do the authors propose to adapt deep learning models to cater to the unique needs of different cities?

Reply: Thanks for your comments. As you pointed out, the original structure of the article has certain issues. To address these problems, we have added interviews with different groups (such as users, property owners, and tourists) to collect design requirements. The specific adjustments are as follows:

1. To better determine the research direction for the renewal of industrial heritage, we will collect opinions and suggestions through interviews based on a review of existing literature. This approach not only helps us identify the shortcomings of current research but also provides valuable insights for future research directions. The interview subjects include experts in industrial heritage conservation, urban planners, historians, community residents, and tourists, from whom we have gathered feedback from a total of 74 participants. We designed the following core questions to guide the interviews:

(1)What do you consider to be the main achievements in the renewal of industrial heritage?

(2)What challenges and problems have you encountered in the design or use process?

(3)In which areas do you think the existing research is lacking?

(4)What suggestions and expectations do you have for future research directions?

(5)How do you think public awareness and participation in industrial heritage can be improved?

By refining and categorizing the information collected from the interviews, we gathered the following user keywords:

(1) Historical and cultural value: historical heritage, cultural identity, cultural preservation, historical atmosphere;

(2) Functionality and practicality: space utilization, ease of use, safety assurance;

(3) Architectural aesthetics: exterior design, visual effects, environmental harmony;

(4)Economic and social benefits: economic feasibility, social impact, enhancement of public services;

(5)Environmental protection and sustainability: sustainable development, environmental conservation;

(6) User experience: smooth pedestrian flow, service quality, and attitude;

(7) Community activity space: educational functions, tourism potential, government support.

2.Based on the results of the interviews and in conjunction with generative artificial intelligence methods, our team, consisting of eight master's and doctoral students, generated multiple design proposals. These proposals utilized different generative AI tools, with each student rigorously adhering to predetermined scenario requirements during the generation process, repeatedly adjusting input prompts and parameters to achieve optimal results across multiple iterations. Finally, we selected two high-quality proposals from each generative AI tool for subsequent evaluation and discussion using the Analytic Hierarchy Process (AHP).

3.We have also made the following adjustments to the originality contributions of the article: The originality and contributions of this research mainly lie in the following aspects: (1)Development of an evaluation system: We designed a multidimensional evaluation system based on the actual needs of industrial heritage renewal and validated its effectiveness through practical research;

(2)Determination of indicator weights: We scientifically determined the weights of various indicators using AHP, providing an objective reference for the application of generative artificial intelligence in industrial heritage renewal;

(3)Empirical analysis: We conducted in-depth empirical analysis of the renewal of industrial heritage in Dalian, demonstrating the application effects and improvement suggestions of generative artificial intelligence tools in real projects.

Response to reviewer 2 comments:

Reviewer#2, Concern # 1: The title of this article is the study of generative artificial intelligence in the application of industrial architectural heritage, is a technical challenge. However, from the article's content, it is a functional application of three intelligent platforms from the known relatively mature ones, and it is not an innovation of a new technology. Does this conflict with the meaning expressed in the title of the article?

Reply: Thanks for your comments. The article utilizes AHP and AIGC to evaluate and renovate the industrial architectural heritage of Dalian. Therefore, We have changed the original title to: "Application and Renovation Evaluation of Dalian's Industrial Architectural Heritage Based on AHP and AIGC."

________________________________________

Reviewer#2, Concern # 2: The article focuses on the three AI platforms' working mechanisms and generation process. This cannot be considered an innovation, but more like a product introduction. The innovation of the article focuses on the post-use evaluation of different programs generated by these platforms, and establishes evaluation impact factors and thresholds. However, the identification of these factors needs to be more strongly exemplified than just applying access from expert interviews and literature, and should it not also be considered and developed in detail respecting the different population aspects such as users, property owners and even tourists?

Reply: Thanks for your comments. The original article only considered and adopted the needs of experts, which cannot fully represent the direction and requirements for the renewal of all industrial heritage buildings. Therefore, we have made the following adjustments: conducting field surveys to collect design requirements from users (including heritage protection experts, urban planners, historians, community residents, and tourists), designing based on these requirements, and finally evaluating the designed proposals to select the best one. The specific adjustments are as follows:

To better determine the future research direction for the renewal of industrial heritage buildings, we will first review existing literature and then collect opinions and suggestions through interviews. This approach not only helps us understand the shortcomings and areas for improvement in current research but also provides valuable insights into future research directions. The interview subjects will cover various fields, including heritage protection experts, urban planners, historians, community residents, and tourists. The interviews will be conducted face-to-face, and we have gathered opinions and suggestions from 74 relevant individuals. Based on the interview goals and subjects, we have designed the following key questions:

(1)What do you consider to be the main achievements in the renewal of industrial heritage?

(2)What challenges and problems have you encountered in the design or use process?

(3)In which areas do you think the existing research is lacking?

(4)What suggestions and expectations do you have for future research directions?

(5)How do you think public awareness and participation in industrial heritage can be improved?

By refining and categorizing the information collected from the interviews, we gathered the following user keywords:

(1) Historical and cultural value: historical heritage, cultural identity, cultural preservation, historical atmosphere;

(2) Functionality and practicality: space utilization, ease of use, safety assurance;

(3) Architectural aesthetics: exterior design, visual effects, environmental harmony;

(4)Economic and social benefits: economic feasibility, social impact, enhancement of public services;

(5)Environmental protection and sustainability: sustainable development, environmental conservation;

(6) User experience: smooth pedestrian flow, service quality, and attitude;

(7) Community activity space: educational functions, tourism potential, government support.

________________________________________

Reviewer#2, Concern # 3: The number of texts in the article is limited to the development of an industrial heritage, with a single sample size and a solidified style. The author collected more than 400 images of industrial heritage from the Internet as data source and model training, but there is no relevant information about how the model is trained, the training process, and the comparison of training results in the article, which is more about the evaluation of the generated scenarios.

Reply: Thanks for your comments. The article focuses on post-use evaluation. We have adjusted the original article structure to include field surveys to collect user design requirements, design based on these requirements, and finally evaluate the designed proposals to select the best one. The revised innovative points and research objectives are as follows:

(1)Construction of the Evaluation System: Designing a multidimensional evaluation system based on the actual needs of industrial heritage renewal and validating its effectiveness through practical research.

(2)Determination of Indicator Weights: Using the Analytic Hierarchy Process (AHP) to scientifically determine the weights of various indicators, providing an objective reference for the application of Generative Artificial Intelligence (AIGC) in industrial heritage renewal.

(3)Empirical Analysis: Conducting an in-depth empirical analysis of the renewal of industrial heritage buildings in Dalian, showcasing the application effects and improvement suggestions of generative AI tools in actual projects.

In addition, regarding the issues you raised about how the model is trained, the training process, and the comparison of training results, we have made the following additions:

During the training process, since Stable Diffusion is a re-generation based on existing models, there are relatively few models related to industrial buildings available online. Therefore, I used the LoRA training software "Cybertron Furnace" for the preprocessing and training of the industrial heritage model. The steps are as follows(Fig 1):

(1)Data Collection: Approximately 400 images of industrial heritage buildings were collected from the internet. To meet the dataset usage requirements, relevant professionals such as designers, master's students, and doctoral students were invited to classify the image types, and duplicates and low-quality images were removed.

(2)Image Preprocessing: The collected images of industrial heritage updates were preprocessed in Photoshop to remove irrelevant information (e.g., watermarks, logos, people).

(3)Model Adjustment: The base model was adjusted to the large model "Stable Diffusion Urban Design Large Model_V7". The invocation phrase was set to "industrial heritage update," and the automatic XYZ and sample preview were set to "architecture."

(4)Training Configuration: In the software, the resolution was set to "512×512", the mode to "focus crop", and TGA to "auto TGA," then the training process was started.

(5)Model Training and Selection: Through multiple batches of training and parameter adjustments, the most suitable LoRA models were selected for subsequent image generation.

**Fig 1. Stable Diffusion drawing process and prompt words**

From the training results, the overall effect achieved the expected goals, but there were differences in performance among the various LoRA models in specific scenarios(Fig 2).

In the "Industrial Heritage Update—1" and "Industrial Heritage Update—2" LoRA models, some scenarios exhibited issues with proportion imbalance and the automatic generation of many irrelevant buildings and water surfaces.

The "Industrial Heritage Update—3" LoRA model performed moderately well overall, but the detail processing was somewhat rough. Especially in Scene 2, the integration of buildings with the environment was average, and the image lacked depth and layering.

The "Industrial Heritage Update—4" and "Industrial Heritage Update—5" LoRA models showed a more natural relationship between buildings and their surrounding environment, with clearer light and shadow relationships and better detail layering and depth.

However, in terms of image richness and stability, the results generated by the "Industrial Heritage Update—5" LoRA model were superior. Therefore, this model was chosen for the subsequent image generation tasks.

**Fig 2. Analysis and evaluation index system of interior design renderings**

________________________________________

Again, the authors are thankful to the anonymous reviewers, editor-in-chief, associate editor, and editorial staff for their time, help, efforts, and support.
