# Supplementary material for: Application and renovation evaluation of Dalian’s industrial architectural heritage based on AHP and AIGC
Source: PLoS One. 2024 Oct 31;19(10):e0312282. doi: 10.1371/journal.pone.0312282 (PMC11527283; doi:10.1371/journal.pone.0312282)
Supplement: Attachment — Submitted filename: Response to Reviewers.doc [file pone.0312282.s002.doc]

Response letter

Journal: PLOS ONE

Title: Application and Renovation Evaluation of Dalian's Industrial Architectural Heritage Based on AHP and AIGC

The authors would like to thank the reviewer for his/ her valuable, detailed, and constructive comments, which helped us further improve the quality of the submitted manuscript. All the comments and suggestions are carefully addressed in the revised manuscript.

The authors tried their level best to address all the queries, and the point-to-point response is given below:

Response to reviewer 1 comments:

Reviewer#1, Concern # 1: There are two small issues that need attention, the originality of the pictures on the Internet, and the grammar after the full text is revised.

Reply: Thanks for your comments. We have taken note of your concerns.

1.In response to the reviewers' concerns about the originality of the images used in the paper, we provide the following explanations regarding the originality of the original images, the images used for LORA training, and the images generated by three AI tools:

(1). Originality of the Original Images

The original images in this study are real-life photographs of the industrial architectural heritage in Dalian, taken by the research team or project participants during field investigations. These images are entirely original visual materials. They authentically reflect the current state of Dalian's industrial architectural heritage and provide accurate visual references for subsequent design research. The research team strictly adhered to relevant laws, regulations, and research ethics during the photography process, ensuring the legality and compliance of the captured content. Therefore, the original images are definitively original, based on first-hand fieldwork, and not derived from or replicated by others.

(2). Originality of the LORA Training Images

For training the LORA model, the data set used was collected and curated independently by the research team, consisting of images related to industrial architectural heritage. These data were sourced from legal public channels, design case libraries, and visual materials created by designers. To ensure the legality and originality of the training images, the research team rigorously screened and processed the data set. Specifically, the images were legally obtained and then re-cropped, labeled, and transformed to align with the project's research objectives, effectively making them secondary creations. The LORA model was trained to optimize generative AI performance in specific scenarios, making the use of these processed images a legitimate practice. The team also adhered to the licensing agreements of open-source communities, such as those for using Stable Diffusion and its plugins, ensuring compliance with copyright laws. Thus, the LORA training images are relatively original and lawful, being reworked and curated from existing resources.

(3). Originality of the AIGC-Generated Images

The originality of the images generated in this study using tools like Stable Diffusion, Mid Journey, and Adobe Firefly is evident in several ways:

- Originality of the Prompt Design: The generation process is based on prompts designed by the research team, tailored to the project’s needs and objectives, representing original creative input.

- Uniqueness of the Generated Images: Although AIGC images are based on large-scale training models, each image is automatically generated by the algorithm, characterized by randomness and uniqueness, with no exact duplication of any existing work.

- Legal Use of Open-Source Models: The tools used in this research are all legal and open-source. According to their licensing agreements, the generated images are permissible for use in non-commercial research projects, ensuring no legal infringement. The team strictly adhered to the usage guidelines for these images, guaranteeing their legal and compliant use.

In conclusion, all images used in this study—whether original, LORA-trained, or AIGC-generated—have undergone thorough originality verification and legality checks, ensuring that no copyright infringement has occurred. The original images were captured during fieldwork, the LORA training images were processed and reworked, and the AIGC-generated images were based on prompts created by the research team, all of which are original. The research team has strictly complied with relevant laws, regulations, and copyright agreements, ensuring the legitimacy and originality of the research outcomes.

2.After the full text was revised, we conducted a thorough grammar check to ensure that the language is accurate, clear, and free of errors. We have made extra efforts to improve the overall flow and readability of the text, addressing any potential grammatical issues.

________________________________________

Response to reviewer 2 comments:

Reviewer#2, Concern # 1: The conclusion of the article is somewhat general and does not align well with the research content. The conclusion should encompass the main contributions of the paper, spotlight the findings of each part of the study, and incorporate the necessary data and indicators.

Reply: Thanks for your comments. We have revised the conclusion based on your suggestions. Below is the updated conclusion:

This study explores the application of generative AI in architectural design, using the renewal of industrial architectural heritage in Dalian as a case study. By testing and comparing three mainstream tools—Stable Diffusion, Mid Journey, and Adobe Firefly—and constructing an evaluation system using the Analytic Hierarchy Process (AHP), this paper systematically analyzes the performance, potential, and limitations of these tools in the context of industrial architectural heritage renewal, while also offering insights into the contributions and future prospects of this research.

In terms of research contributions, the study first established an evaluation system tailored to industrial architectural heritage renewal. By analyzing national policy documents, highly cited literature, and incorporating expert consultation and field research, four primary evaluation indicators were proposed: architectural renovation, environmental aesthetics, economic benefits, and socio-cultural value. These were further divided into 16 secondary indicators, providing a scientific basis for generating and evaluating design schemes. Analysis of expert questionnaire scores revealed that "architectural aesthetic continuity" and "improvement of quality of life" had the highest weights in the project, with values of 0.380 and 0.363, respectively, indicating that maintaining the historical continuity of architectural aesthetics and enhancing the quality of life for surrounding residents are core demands in the renewal of industrial heritage. In the comparative analysis of generative AI tools, Stable Diffusion scored the highest in the indicators of "architectural renovation" and "socio-cultural value," with scores of 6.380 and 6.921, respectively, and an overall score of 6.3. Mid Journey showed balanced performance in "environmental aesthetics" and "economic benefits" but scored lower in "architectural renovation," with scores of 5.072 and 4.202. Adobe Firefly excelled in speed and ease of use, making it suitable for quickly generating concept images.

Despite the significant advantages demonstrated by generative AI tools in this study, certain limitations remain. First, these tools still struggle with understanding human emotions and cultural contexts, which may lead to design schemes lacking in emotional resonance and cultural symbolism. Second, the randomness and uncontrollability of generated results are challenges that designers must address, especially when using Mid Journey, where multiple iterations and selections are required to achieve the desired outcome. Additionally, the use of generative AI tools places higher demands on designers' skills, requiring them to not only master traditional design theory but also become proficient in leveraging these new tools for scheme optimization. As generative AI technology continues to develop, its application prospects in the renewal of architectural heritage are broad, but further technological optimization and enhanced collaboration between designers and AI are necessary.

Overall, generative AI tools provide new approaches and methods for the renewal of industrial architectural heritage, significantly simplifying the design process while improving efficiency and quality. Although some technical and application limitations still exist, with ongoing advancements, generative AI is poised to play an increasingly significant role in the design field, driving innovation and development in the industry.

________________________________________

Again, the authors are thankful to the anonymous reviewers, editor-in-chief, associate editor, and editorial staff for their time, help, efforts, and support.
