# Supplementary material for: Application and renovation evaluation of Dalian’s industrial architectural heritage based on AHP and AIGC
Source: PLoS One. 2024 Oct 31;19(10):e0312282. doi: 10.1371/journal.pone.0312282 (PMC11527283; doi:10.1371/journal.pone.0312282)
Supplement: Attachment — Submitted filename: Response to Reviewers.doc [file pone.0312282.s003.doc]

Response letter

Journal: PLOS ONE

Title: Application and Renovation Evaluation of Dalian's Industrial Architectural Heritage Based on AHP and AIGC

The authors would like to thank the reviewer for his/ her valuable, detailed, and constructive comments, which helped us further improve the quality of the submitted manuscript. All the comments and suggestions are carefully addressed in the revised manuscript.

The authors tried their level best to address all the queries, and the point-to-point response is given below:

Response to reviewer 1 comments:

Reviewer#1, Concern # 1: Generally speaking, we try to avoid large areas of text descriptions when drawing pictures. This is because the display effect is not good. For example, in Figure 1, there are a lot of notes at the bottom. They can be merged into the text or annotated after the figure name.

Reply: Thanks for your comments. We understand that large areas of text can affect the overall display quality of illustrations. Therefore, we have merged the annotations into the main text to improve clarity and presentation.

________________________________________

Reviewer#1, Concern # 2:The research background in the introduction needs further clarification. First of all, the design process is completely different in different social systems. In essence, this article is a comparative analysis of the effects, evaluation, and tools of several platforms of the design renderings of Dalian Industrial Heritage. This should be very helpful to designers in the early stages, especially when meeting the needs of clients and owners, and can improve efficiency. However, whether it is the final solution and will help the transformation of industrial heritage remains to be discussed. After the design renderings are completed, they are still mainly completed by structural engineers and builders, and they may be modified. Therefore, it should be emphasized at which stage artificial intelligence helps designers or practitioners in generating design renderings.

Reply: Thanks for your comments. We can agree with the final solution you proposed, but whether it contributes to the transformation of industrial heritage remains to be discussed. Based on your feedback, we emphasize that the early stages of conceptual design and rendering play a significant role. Below is the revised introduction:

The traditional architectural design process is complex, requiring designers to independently complete a large amount of work. During the design process, they must consider solutions that meet legal regulations, ecological principles, aesthetic creativity, plant configuration, traffic flow, materials, and costs. In terms of visual representation, the process involves conceptualization, sketching, modeling, and rendering. These tasks demand significant human and material resources. Due to the complexity, even minor adjustments often require designers to repeat certain parts of the design process. Additionally, clients usually ask designers to provide multiple design options for a project, which increases the workload. As a result, this design process and market environment lower the efficiency of design projects, and the overwhelming tasks limit the designer's creativity from being fully expressed .

The traditional methods present several key issues that need improvement: 1. Time and efficiency, 2. Repetitive tasks, 3. The demand for multiple design options, and 4. Substantial human and material resource investment. The emergence of Generative AI (AIGC) has replaced many traditional design processes, gradually transforming the designer's role from "creator" to "decision-maker." AIGC provides a new approach to design, effectively addressing these issues.

AIGC's characteristics make it particularly useful in assisting with the early, heavy, and repetitive stages of conceptual design and rendering. Specific improvements include: 1. Automated generation of initial concepts: Designers only need to input keywords to quickly generate multiple preliminary designs, shortening the design cycle. 2. Reducing repetitive tasks: AIGC can automate many traditional design steps, such as concept generation, color matching, and structure optimization, allowing designers to adjust only the parameters. 3. Efficient multi-option generation: AIGC enables designers to quickly generate multiple options for clients to choose from, improving client satisfaction. 4. Optimizing resource allocation: AIGC tools typically integrate various AI technologies, such as deep learning and edge detection algorithms, to quickly generate high-quality renderings, reducing reliance on specialized software and manpower. This approach transforms conceptualization directly into visualization, replacing many traditional design processes. By combining AI thinking with the designer’s creative thought process, the design cycle is significantly shortened.

To comprehensively evaluate the practical application of generative AI tools, this study has developed a multidimensional evaluation system for the renewal of industrial architectural heritage. This system includes specific indicators such as design quality, generation efficiency, ease of use, cultural and emotional understanding, innovation, and practicality. Using the Analytic Hierarchy Process (AHP) and weighted scoring methods, we conducted a comprehensive evaluation of three generative AI tools to ensure the analysis is thorough and scientific. Through an in-depth empirical analysis of the renewal of industrial architectural heritage in Dalian, the application and improvement suggestions of generative AI tools in real projects are demonstrated.

In summary, the innovative and original contributions of this paper are: 1. Construction of an evaluation system: A multidimensional evaluation system was designed based on the actual needs of industrial heritage renewal, and its effectiveness was verified through practical research. 2. Determination of indicator weights: The Analytic Hierarchy Process was used to scientifically determine the weight of each indicator, providing objective reference points for the application of generative AI in industrial heritage renewal. 3. Empirical analysis: An in-depth empirical analysis was conducted through the renewal of Dalian's industrial architectural heritage, demonstrating the application of generative AI tools in real projects and providing improvement suggestions.

________________________________________

Reviewer#1, Concern # 3: The literature review introduces the contents of different AI generation software. However, a literature review does not need introductory text. It should focus more on what scholars have studied in such applied research and what problems remain to be solved. In this study, what did the author try to solve?

Reply: Thanks for your comments. We understand that the literature review should focus more on relevant research rather than introductory content. In the revised section, we will highlight key studies that address the application of AI generation software in design, as well as unresolved challenges in the field. We will also clarify the specific problem this study aims to solve, emphasizing the role of AI tools in optimizing the design process and enhancing decision-making in industrial architectural heritage projects.Below is the revised Literature Review:

The protection and renewal of industrial heritage have gained increasing attention in the field of architecture, as these heritages hold significant historical, cultural, and social value. However, these processes face numerous challenges, such as structural degradation, the need for functional adaptation, and the difficulty of integrating modern requirements while maintaining historical integrity. Traditional methods are often labor-intensive, time-consuming, and costly.Therefore, there is an urgent need for innovative approaches to simplify these processes and improve protection outcomes, as emphasized by existing research.

Generative AI (AIGC), as a cutting-edge solution, offers tools that automate and enhance various stages of architectural design. AIGC refers to a class of algorithms capable of creating new content based on input data. In the context of architectural design, these algorithms can generate alternative design options, simulate structural impacts, and provide detailed visual effects. Although the application of AIGC in industrial heritage is still a relatively novel field, it has shown significant potential in addressing the pain points of traditional preservation methods.

One of the main advantages of generative AI is its ability to handle complex design tasks traditionally performed by humans. The traditional architectural design process involves multiple stages, including conceptualization, sketching, modeling, and rendering, each requiring substantial human intervention. This is not only time-consuming but also prone to human error. Generative AI tools can automate these tasks, reducing the time and effort needed to produce high-quality designs. Research by Wang et al. (2021) and Chen (2022) shows that these tools can significantly shorten design cycles while improving design quality and efficiency.

Stable Diffusion, a generative AI tool based on diffusion models, has demonstrated strong control and flexibility in generating architectural designs. Researchers widely agree that this tool excels in producing high-resolution images and handling intricate details. A study by Cao et al. (2024) on the application of Stable Diffusion in architectural design highlights its ability to significantly reduce the workload for designers when generating complex architectural structure diagrams. Specifically, in the visualization of architectural heritage, Stable Diffusion, through its denoising process, can gradually generate clearer and more detailed renderings. Zhang et al. (2023) point out that the tool’s output can be further optimized through plugins, such as using Control Net’s Canny edge detection algorithm to maintain the accuracy of generated images. However, despite its technical excellence, Stable Diffusion has some limitations. According to Hu et al. (2021), the tool requires substantial training data and computing power, and without fine-tuning on specific datasets, the generated renderings may deviate from actual design requirements.

Based on the features of Stable Diffusion and the project’s requirements, the model can be fine-tuned using LORA to improve its performance. Additionally, plugins such as Control Net’s Canny (edge detection algorithm) and Depth (depth algorithm) can enhance image details, and the Tiled Diffusion and Tiled VAE plugins can improve image resolution.

MidJourney, driven by Generative Adversarial Networks (GANs), is an AI design tool widely used in the early stages of conceptual creation in architectural design due to its ability to generate highly artistic and visually impactful images. Goodfellow et al. (2014) laid the theoretical foundation for GANs in their study on ‘Generative Adversarial Nets,’ explaining the basic architecture and training principles of GANs, where the generator learns to create data from random noise, and the discriminator distinguishes real data from generated data. Karras (2017) introduced a progressive training method that significantly improved the quality and resolution of generated images by training GANs at multiple resolution stages. In his later research, Karras (2020) proposed the StyleGAN architecture, which better controls the features of generated images, such as textures and colors.

In practice, Caires et al. (2023) suggest that combining design thinking methods with AIGC enables users to generate innovative and artistically expressive design concepts during the ideation phase, enhancing the design experience. Jiang et al. (2023) note that MidJourney’s adversarial generation mechanism can quickly produce diverse design options, providing architects with a wealth of inspiration. Petráková et al. (2023) also mention that the tool demonstrates excellent composition and color coordination abilities in generating conceptual design drawings, making it highly advantageous in the early stages of architectural design.

Adobe Firefly, a generative AI tool integrated into Adobe Creative Cloud, is mainly used for quickly generating prototype designs and conceptual plans. Through deep learning and edge detection algorithms, it can generate efficient design options in a short amount of time and is known for its ease of use. A quantitative analysis by Poredi et al. (2024) shows that Adobe Firefly is a highly efficient AIGC tool that is easy to learn and interact with, making it ideal for generating multiple design options in the early stages of design, thereby significantly reducing design time. Research by Persson et al. (2023) and Cook et al. (2024) indicates that this tool can be used as a standalone web application or directly within applications such as Photoshop and Illustrator, seamlessly integrating Firefly’s generative features into familiar working environments. This integration allows users to harness AI technology to enhance the creative process, improving productivity. Haze et al. (2023) believe that Adobe Firefly’s ease of use and professional capabilities help enhance users' learning and creativity. Moreover, Firefly is integrated into the Adobe Express platform, providing users with a suite of user-friendly tools for creating professional-quality visual content.

Despite its significant advantages, generative AI still faces certain limitations and challenges. One of the main challenges is incorporating cultural and emotional elements into AI-generated designs. Current AI tools often lack a comprehensive understanding of the cultural and emotional significance of heritage sites, which may result in aesthetically pleasing designs that are culturally inappropriate. Researchers such as Li et al. (2023) are working to integrate cultural heritage data into AI algorithms to enhance their cultural and emotional sensitivity. This includes training AI models on datasets containing cultural and historical information to generate designs that are both visually appealing and culturally relevant. Another challenge is that applying generative AI in industrial heritage projects requires interdisciplinary collaboration. Preservation and renewal projects typically involve multiple stakeholders, including architects, historians, engineers, and community members. Effective communication and collaboration are crucial to the success of such projects. Generative AI tools facilitate this collaboration by providing detailed visualizations and simulations, promoting the sharing and discussion of ideas among stakeholders, ensuring that all viewpoints are considered, and the final design meets the needs and expectations of all parties.

In addition, data quality and availability are issues that need to be addressed. Generative AI models require large amounts of high-quality data to function effectively, including data on existing structures, materials, and historical records. In many cases, this data may be incomplete or unavailable, limiting the effectiveness of AI tools. Researchers are exploring advanced data collection techniques and developing more complex AI models capable of handling incomplete or imperfect data to address this issue.

Overall, generative AI offers significant potential benefits for the preservation and renewal of industrial architectural heritage. By automating many tasks traditionally performed by human designers, these tools can reduce the time and cost of preservation projects, improve design quality and precision, and provide abundant creative inspiration. Additionally, the ability of generative AI to quickly and efficiently generate multiple design alternatives helps ensure the best solution is found for each project.

________________________________________

Reviewer#1, Concern # 4: The CGAN algorithm was mentioned in the literature review. Why didn't the author directly look for a literature review on generative design related to CGAN?

Reply: Thanks for your comments. We understand your concerns.Although GANs hold a significant position in generative design, this study is not limited to a single generative algorithm. Instead, it explores the application of various AI generative tools in architectural design. We selected several representative generative AI tools, such as Stable Diffusion, MidJourney, and Adobe Firefly, each of which has unique advantages in practical applications.

The focus of the literature review is on the specific effects of these tools in the design field, rather than a comprehensive analysis of any particular algorithm. Therefore, the study is not confined to literature on GANs but takes a broader perspective to discuss the practical contributions of generative AI in architectural heritage preservation and design. This also helps to better analyze the potential of these tools in interdisciplinary collaboration and optimizing design processes. Although we did not conduct a separate literature review specifically on GANs algorithms, our analysis of specific software applications effectively covers the practical use of GANs in generative design,such as MidJourney.

________________________________________

Reviewer#1, Concern # 5: What is the basis for distributing questionnaires to 10 experts? What are the representative characteristics of the experts?

Reply: Thanks for your comments. We appreciate your concerns and would like to provide an explanation of our criteria for selecting experts:

（1）Professional Background: All invited experts have over five years of experience in the fields of industrial heritage preservation, architectural design, urban planning, and related areas. This includes architects, structural engineers, heritage scholars, and urban planners. Their extensive practical experience enables them to provide expert insights into the renovation and transformation of industrial heritage.

（2）Academic Qualifications: Most members of the expert team hold senior titles (such as professor-level senior engineers, researchers, etc.) and have published research related to industrial heritage preservation in authoritative domestic and international journals. They have also participated in various industrial heritage renovation projects across different countries or regions.

（3）Geographic Representation: To ensure diversity and comprehensiveness in the evaluation results, the invited experts come from different regions. This reflects a wide range of perspectives and needs concerning industrial heritage renovation.

________________________________________

Again, the authors are thankful to the anonymous reviewers, editor-in-chief, associate editor, and editorial staff for their time, help, efforts, and support.
